# SIRT1 Mediates FOXA2 Breakdown by Deacetylation in a Nutrient-Dependent Manner

**DOI:** 10.1371/journal.pone.0098438

**Published:** 2014-05-29

**Authors:** Rogier van Gent, Claudio Di Sanza, Niels J. F. van den Broek, Veerle Fleskens, Aukje Veenstra, Gerdine J. Stout, Arjan B. Brenkman

**Affiliations:** 1 Center for Molecular Medicine, Department of Molecular Cancer Research, Section Metabolic Diseases, University Medical Center Utrecht, Utrecht, The Netherlands, and Netherlands Metabolomics Centre, Leiden, The Netherlands; 2 Erasmus Medical Center Rotterdam, Department of Gastroenterology and Hepatology, Rotterdam, The Netherlands; 3 University Medical Center Utrecht, Department of Cell Biology, Utrecht, The Netherlands; University of South Florida, United States of America

## Abstract

The Forkhead transcription factor FOXA2 plays a fundamental role in controlling metabolic homeostasis in the liver during fasting. The precise molecular regulation of FOXA2 in response to nutrients is not fully understood. Here, we studied whether FOXA2 could be controlled at a post-translational level by acetylation. By means of LC-MS/MS analyses, we identified five acetylated residues in FOXA2. Sirtuin family member SIRT1 was found to interact with and deacetylate FOXA2, the latter process being dependent on the NAD^+^-binding catalytic site of SIRT1. Deacetylation by SIRT1 reduced protein stability of FOXA2 by targeting it towards proteasomal degradation, and inhibited transcription from the FOXA2-driven *G6pase* and *CPT1a* promoters. While mutation of the five identified acetylated residues weakly affected protein acetylation and stability, mutation of at least seven additional lysine residues was required to abolish acetylation and reduce protein levels of FOXA2. The importance of acetylation of FOXA2 became apparent upon changes in nutrient levels. The interaction of FOXA2 and SIRT1 was strongly reduced upon nutrient withdrawal in cell culture, while enhanced Foxa2 acetylation levels were observed in murine liver *in vivo* after starvation for 36 hours. Collectively, this study demonstrates that SIRT1 controls the acetylation level of FOXA2 in a nutrient-dependent manner and in times of nutrient shortage the interaction between SIRT1 and FOXA2 is reduced. As a result, FOXA2 is protected from degradation by enhanced acetylation, hence enabling the FOXA2 transcriptional program to be executed to maintain metabolic homeostasis.

## Introduction

The mammalian Forkhead transcription factor FOXA2 is a key regulator of hepatic energy metabolism in mammals [1]. Besides its role in regulating liver and other endoderm-derived organ specification during embryonic development [2], in post-natal life Foxa2 controls essential metabolic processes including glucose metabolism [3], [4], bile acid homeostasis [5] and lipid oxidation [6], [7]. Hepatocyte-specific Foxa2 conditional knockout mice showed impaired glucose homeostasis during the fasting response [3] and Foxa2 heterozygous mice displayed increased adiposity and impaired glucose uptake when challenged with a high fat diet [6], [8]. In *C. elegans* and *D. melanogaster*, orthologues of FOXA are involved in gut development, illustrating the importance of these proteins for organ specification throughout metazoan evolution [9], [10]. Interestingly, the nematode FOXA orthologue PHA-4 was found to control dietary restriction (DR)-mediated lifespan extension in *C. elegans* [11], suggesting a conserved role for FOXA transcription factors in energy homeostasis and metabolism, although the evidence for the involvement of mammalian FOXA2 in DR-mediated lifespan extension is still absent.

Metabolic processes controlled by Foxa2, such as glucose and lipid homeostasis, are often deregulated in metabolic syndromes such as diabetes and obesity [12], [13]. Therefore, it is of great importance to investigate and understand how changes at a metabolic level functionally affect FOXA2-mediated transcriptional regulation of metabolism. Recent reports identified several post-translational modifications which are important in regulating Foxa2 metabolic function and cellular localization. Insulin-activated AKT2/PKB phosphorylates Foxa2 on a specific threonine (T156) adjacent to the DNA binding domain, resulting in nuclear exclusion and subsequent reduced transcriptional output by Foxa2 [13], [14], although these findings remain controversial [3], [15]. IKKα kinase, a downstream target of the pro-inflammatory cytokine TNFα, was also reported to suppress the FOXA2 transcriptional program by phosphorylating two serines (Ser107/111) adjacent to the DNA binding Forkhead domain [16].

Acetylation was recently proposed as a key post-translational mechanism by which transcription factors involved in metabolism are controlled in a nutrient-dependent manner [17], [18]. Acetylation could therefore be a critical post-translational modification to regulate metabolic activity of FOXA transcription factor family members. Indeed, FOXA transcription factors were found to synergize with acetyltransferase p300 towards gene activation [19], [20]. Moreover, FOXA1 was shown to be acetylated by p300 [21]. In contrast to acetyltransferases which add acetylation moieties to proteins, protein deacetylation is carried out by several deacetylases. These are grouped to either class I and II histone deacetylases (HDACs) or class III NAD^+^-dependent deacetylases, or sirtuins, which are activated upon changes in energy levels as manifested by an increase in the cellular NAD^+^/NADH ratio. Among the sirtuin family members, SIRT1 has the broadest range of substrates and affects the largest number of physiological pathways, including glucose and lipid metabolism [22], [23]. Because of its NAD^+^ dependency [24], SIRT1 connects the energy demands of a cell to its metabolic regulation via deacetylation of metabolic transcription factors and co-activators. Therefore, sirtuins may be involved in energy-dependent regulation of FOXA transcription factors by controlling their level of acetylation.

Since FOXA2 functions as a metabolic gauge, we hypothesized that FOXA2 could be regulated in a nutrient-dependent manner by means of acetylation. By using LC-MS/MS, we identified several acetylation sites on FOXA2 which are located adjacent to the DNA binding domain of the protein. Acetylation of FOXA2 was dependent on SIRT1 which was found to interact with and deacetylate FOXA2. Interestingly, the acetylation level of FOXA2 and the interaction of FOXA2 with SIRT1 were nutrient-dependent. Moreover, acetylation protected FOXA2 from proteasomal degradation to sustain transcriptional activity of FOXA2 on its target genes. Taken together our data point to a model in which FOXA2 stability is regulated by SIRT1-mediated deacetylation in a nutrient-dependent manner towards the maintenance of whole-body metabolic homeostasis.

## Materials and Methods

### Cell culture and transfection

All cell lines were obtained from ATCC. Human embryonic kidney 293T cells (HEK293T cells) were maintained in DMEM containing 1 g/L glucose (Glutamax, Gibco) supplemented with 10% FCS and antibiotics. Human bone osteosarcoma epithelial cells (U2OS) and the human hepatoma HepG2 cell line were maintained in DMEM containing 4.5 g/L glucose (Glutamax, Gibco) supplemented with 10% FCS and antibiotics. In experiments in which cells were starved, cells were washed thrice with PBS prior to culture in glucose-free medium (Gibco) without FCS. All cell lines were cultured in a humidified atmosphere containing 5% CO_2_ at 37°C. Transient transfection of HEK293T cells was performed with FuGene 6 (Roche, Basel, Switzerland) according to manufacturer's instructions, while HepG2 cells and U2OS cells were transiently transfected with polyethylenimine (PEI) (Polysciences) at a ratio of 1 µg DNA to 4 µl PEI. Transfection of siRNA was performed with Oligofectamine (Invitrogen) according to manufacturer's instructions.

### Constructs

Wild type pcDNA3-FLAG-FOXA2 was synthesized by GenScript (Piscataway, NJ, USA). pRSV-MYC-SIRT1 and pRSV-MYC-SIRT1 H363Y were kind gifts from Prof. P. Coffer. pGL3-G6pase-Luc (*G6pase* promoter [−1227 to +57]) was a kind gift from Dr. D. Schmoll [25] and pGL3-CPT1a-Luc (*Cpt1a* promoter [−2432 to +1]) was a kind gift from Prof. M. Negishi [26]. Mutation of multiple acetylation sites to generate the 12K-R FLAG-FOXA2 mutant was performed by mutating (from lysine (K) to arginine (R)) the wild type pcDNA3-FLAG-FOXA2 construct by site-directed mutagenesis (SDM) for the following residues: K6, K259, K264, K274, K275 (identified by our LC/MS-MS analysis) and K149, K226, K229, K253, K256, K365, K399 (putative acetylation sites).

### Luciferase assay and siRNA

To assess FOXA2-mediated transcriptional activity on either the glucose-6-phosphatase (*G6pase*) or carnitine palmitoyltransferase 1a (*CPT1a*) promoter, pcDNA3-FLAG-FOXA2, pTK-*Renilla* and pGL3-G6pase-Luc or pGL3-CPT1a-Luc (and pRSV-MYC-SIRT1 when indicated) were transfected in HEK293T cells and after 48 hours samples were assayed for luciferase activity. To study the effect of knockdown of endogenous SIRT1 expression on FOXA2 transcriptional activity in HEK293T cells, two rounds of SIRT1 knockdown (8-hour interval) were performed with Oligofectamine (Invitrogen) according to manufacturer's instructions, followed by transfection of pcDNA3-FLAG-FOXA2, pGL3-G6pase-Luc and pTK-*Renilla* after 16 hours. Forty-eight hours after this final transfection, samples were assayed for luciferase activity. Knockdown of SIRT1 was performed using ON-TARGETplus SIRT1 siRNA SMARTpool, while ON-TARGETplus control siRNA SMARTpool (Thermo Scientific, Lafayette, CO, USA) was used as a siRNA transfection control. Luciferase assays were performed with the Dual-Luciferase Reporter Assay System according to manufacturer's instructions (Promega). pTK-*Renilla* was used for normalization of promoter-driven luciferase expression.

### Antibodies and reagents

The following antibodies were used for confocal imaging: mouse-anti-FLAG (M2, F1804) (Sigma), rabbit-anti-SIRT1 (D739, #2493) (Cell Signaling), goat-anti-FOXA2 (M-20, sc-6554) (Santa Cruz Biotechnology). For Western Blot analyses the following antibodies were used: rabbit-anti-beta-actin (ab8224) (Abcam), mouse-anti-FLAG (M2, F1804) (Sigma), mouse-anti-MYC (9E10, ab32) and rabbit-anti-acetyl-lysine-HRP (ab23364) (Abcam), rabbit-anti-FOXA2 (WRAB-1200) (Seven Hills Bioreagents), rabbit-anti-SIRT1 (D739, #2493) (Cell Signaling), anti-ubiquitin-HRP (BML-PW0150) (Enzo Lifesciences). Nicotinamide (NAM), Trichostatin A (TSA), cycloheximide and MG132 were purchased from Sigma. Concentration and duration of exposure of cells to each of these reagents is indicated for each experiment.

### Immunoprecipitation and Western Blot analyses

To assess the acetylation status of FOXA2, wild type pcDNA3-FLAG-FOXA2 was ectopically expressed in HEK293T cells. After 48 hours, cells were lysed in RIPA buffer containing 1% Triton X-100, 0.5% sodium deoxycholate, 20 mM Tris-HCl pH8.0, 167 mM NaCl, 10 mM EDTA, 0.1% SDS and Complete protease inhibitors (Sigma). FLAG-FOXA2 was immunoprecipitated using FLAG-M2 agarose beads (Sigma) at 4°C for 2 hours. Beads were subsequently washed three times in RIPA buffer. To minimize co-elution of the heavy and light chains of the anti-FLAG antibodies, the beads were resuspended in non-reducing sample buffer (containing: 125 mM Tris-HCl pH6.8, 4% SDS, 20% glycerol and 0.004% bromophenol blue) and heated at 95°C for 5 minutes. Subsequently, the supernatant containing dissociated and immunoprecipitated FLAG-FOXA2 was transferred to a new vial and the proteins were reduced by adding DTT to a final concentration of 50 mM. Immunoprecipitated proteins were analyzed by sodium dodecyl sulfate polyacrylamide gel electrophoresis (SDS-PAGE) followed by Western Blot analyses. Protein acetylation was detected using an anti-acetyl-lysine-HRP antibody (ab23364) (Abcam). Subsequently, immunoprecipitated levels of FOXA2 were assessed by anti-FLAG antibodies (M2, F1804) (Sigma).

To assess the protein-protein interaction by co-immunoprecipitation, FLAG-FOXA2 and wild type or H363Y mutant MYC-SIRT1 were ectopically expressed in HEK293T cells. After 48 hours, cells were lysed in SIRT buffer, containing 1% Triton X-100, 5% glycerol, 50 mM Tris-HCl pH7.5, 100 mM NaCl, 5 mM EDTA and Complete protease inhibitors (Roche, Basel, Switzerland). FLAG-FOXA2 was immunoprecipitated using FLAG-M2 beads (Sigma) for 2 hours. Beads were subsequently washed three times in SIRT buffer and immunoprecipitated proteins were analyzed by SDS-PAGE after reduction in sample buffer, followed by Western Blot analyses. Presence of SIRT1 was detected by anti-MYC (9E10, ab32) (Abcam) or anti-SIRT1 (D739, #2493) (Cell Signaling) antibodies while FOXA2 was detected by anti-FLAG antibodies (M2, F1804) (Sigma).

### Quantitative RT-PCR

To assess the effect of NAM treatment on the levels of *FOXA2* mRNA, HEK293T cells transfected with wild type pcDNA3-FLAG-FOXA2, were cultured for 16 hours with 20 mM NAM of vehicle control and relative *FOXA2* mRNA levels were determined by quantitative RT-PCR. RNA was isolated from the cells using a Machery-Nagel NucleoSpin RNA II kit (Bioké, Leiden, The Netherlands) and quantified using a Nanodrop ND-1000 (Wilmington, DE, USA). cDNA was prepared from total RNA using an iScript cDNA Synthesis Kit from Bio-Rad (Bio-Rad Laboratories, Stanford, CA, USA) and gene expression assays were performed according to manufacturer's protocol (SYBR Select Master Mix; Life Technologies Bleiswijk, The Netherlands) to measure mRNA levels of *FOXA2* as the gene of interest, and *18 S* as the housekeeping gene using the following primers: FOXA2-forward 5′-TGGAGCAGCTACTATGCAG-3′, FOXA2-reverse 5′-CGTGTTCATGCCGTTCATC-3′; 18 S-forward 5′-AGTCCCTGCCCTTTGTACACA-3′ and 18 S-reverse 5′-CGATCCGAGGGCCTCACTA-3′.

### Mass spectrometry

Wild type FLAG-FOXA2 was ectopically expressed in HEK293T cells. When indicated, cells were cultured for 16 hours prior to cell lysis in 20 mM NAM and 5 µM TSA to inhibit endogenous deacetylase activity. Cells were lysed in RIPA buffer, immunoprecipitated with FLAG-M2 agarose beads and reduced in sample buffer, as described above. Subsequently, immunoprecipitated wild type FLAG-FOXA2 was run on NuPAGE Bis-Tris Mini Gels (Invitrogen, Carlsbad, USA) according to manufacturer's instructions. Bands were excised from the gel, reduced with DTT (0.5 mM; MP Biomedicals), alkylated with iodoacetamide (54 mM; Sigma), and digested with trypsin (Roche), chymotrypsin (Roche) or V8-DE (Roche) as described [27]. A second round of digestion was performed on the gel fragments with elastase (Sigma) to improve protein coverage by MS. Samples were subjected to nanoflow LC (Eksigent) using C18 reverse phase trap columns (Synergi 4 µm Hydro-RP 80 Å, Phenomenex; column dimensions 2 cm×100 µm, packed in-house) and subsequently separated on C18 analytical columns (ReproSil-Pur 120 C18-AQ, 5 µm, Dr. Maisch GmbH; column dimensions, 20 cm×50 µm; packed in-house) using a linear gradient from 0 to 40% buffer B (buffer A = 5% (v/v) acetonitrile, 0.1% formic acid (v/v); buffer B = 95% (v/v) acetonitrile, 0.1% formic acid (v/v)) in 60 min at a constant flow rate of 150 nl/min. Column eluate was directly coupled to a LTQ-Orbitrap-XL mass spectrometer (Thermo Scientific) operating in positive mode, using lock spray internal calibration. Data were processed and subjected to database searches using Proteome Discoverer software (Thermo Scientific) or Mascot software (Matrix Science) against non-redundant SwissProt and NCBI database, with a 10 ppm mass tolerance of precursor and 0.5 Da for the fragment ion.

### Localization studies

HepG2 cells were grown on Poly-L-Lysine (Sigma) coated microscope slides, fixed in PBS containing 4% paraformaldehyde (Merck, Nottingham, United Kingdom), and permeabilized in PBS containing 0.25% Triton X-100 (Sigma). Cells were stained with indicated SIRT1 (D739, #2493) (Cell Signaling) and FOXA2-specific antibodies (M-20, sc-6554) (Santa Cruz Biotechnology) in PBS containing 0.05% Tween, 1% BSA (Sigma) and 10% normal donkey serum (Jackson Immunoresearch). Subsequently, cells were extensively washed in PBS containing 0.05% Tween and mounted in Mowiol 4-88 (Sanofi-Aventis) containing DAPI. Cells were analyzed with a 63× objective on a Zeiss LSM 710 confocal microscope (Oberkochen, Germany).

### Proximity Ligation Assay [28]

Cells were grown (and transfected in the case of U2OS cells) as indicated on Poly-L-Lysine-coated microscope slides (Sigma). Cells were fixed in PBS containing 4% Paraformaldehyde (Merck, Nottingham, United Kingdom) for 15 minutes and permeabilized with PBS containing 0.25% Triton X-100 (Sigma) for 10 minutes. Slides were blocked in PBS containing 0.05% Tween and 10% normal donkey serum (Jackson Immunoresearch), and incubated with indicated antibodies against SIRT1 (D739, #2493) (Cell Signaling) and FLAG (M2, F1804) (Sigma), (in transfected U2OS cells), or SIRT1 (D739, #2493) (Cell Signaling) and FOXA2 (M-20, sc-6554) (Santa Cruz Biotechnology) (in HepG2 cells). Cells were subsequently incubated with Duolink II PLA probes (Olink Biosciences) and stained according to manufacturer's protocol. Cells were analyzed with a 63× objective on a Zeiss LSM 710 confocal microscope (Oberkochen, Germany). In experiments in which HepG2 cells were starved, cells were washed thrice with PBS. Subsequently, cells were cultured for 2 or 4 hours in glucose-free medium (Gibco) without FCS prior to fixation.

### Murine livers and endogenous Foxa2 immunoprecipitation

Wild type Balb/c mice were either fed *ad libitum* or were restricted from food intake by limiting the chow to 25% of *ad libitum* levels for 36 hours. Mice were sacrificed, and livers were extracted and stored at −80°C. Whole cell lysates of these livers were generated by homogenizing 50 mg of each liver in RIPA buffer with 0.5 mm Zirconium Oxide beads by Bullet Blender (Next Advance, USA). Immunoprecipitation of Foxa2 from the supernatant of these homogenates was performed by rabbit-anti-FOXA2 antibody (WRAB-1200) (Seven Hill Bioreagents), pre-coupled to protein G sepharose beads (GE healthcare) for 2 hours at 4°C. Beads were subsequently washed three times in RIPA buffer. Elution of the immunoprecipitated proteins and assessment of Foxa2 and its acetylation levels was similar as described above for immunoprecipitation of FLAG-FOXA2.

This study was carried out following ethical approval for studies on laboratory animals by the Dierexperimentencommissie (DEC) of the UMC Utrecht. All efforts were undertaken to minimize suffering of the animals.

### Statistics

Protein expression levels were measured by densitometry using Image J (http://rsbweb.nih.gov/ij/). Protein levels were expressed relative to actin protein levels after subtraction of background intensity. Luciferase data are shown as mean +SEM from 3 independent experiments as analyzed by two-tailed Student's t-test with statistical significance *P*<0.05.

## Results

### FOXA2 is acetylated at multiple residues

**Figure 1:**
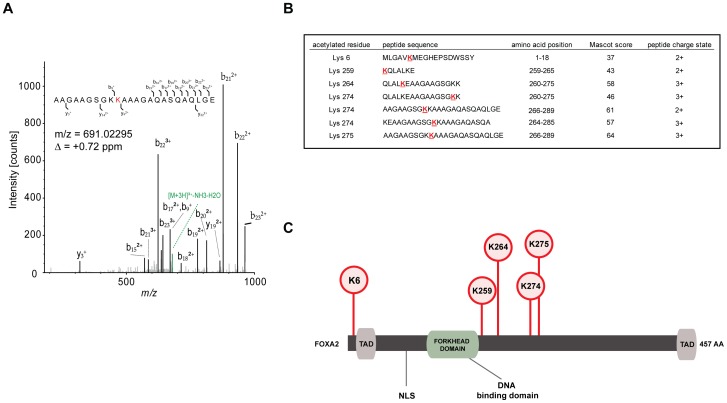
FOXA2 is an acetylated protein. (A) LC-MS/MS spectrum and the deduced peptide sequence of FOXA2 acetylation at lysine K275 (in red). (B) Peptides containing five *in vivo* FOXA2 acetylation sites which were identified by LC-MS/MS analyses. FLAG-FOXA2 was expressed in human kidney HEK293T cells, purified and digested with proteases. Peptides were separated and enriched by C18 analytical columns and subsequently subjected to LC-MS/MS. Acetylated residues in red and underlined. (C) Schematic representation of the FOXA2 protein structure. Markers illustrate the localization of the five acetylation sites and colored boxes represent functional domains. TAD, *Trans Activation Domain*. NLS, *Nuclear Localization Sequence*.

The FOXA2 transcription factor is an essential player in liver metabolism, although the precise regulation of this transcription factor is not completely understood. We hypothesized that post-translational modifications (PTMs) such as acetylation could affect and differentially regulate the FOXA2 metabolic program. To investigate whether FOXA2 is an acetylated protein, Liquid Chromatography-tandem Mass Spectrometry (LC-MS/MS) analyses were performed. To this end, FLAG-FOXA2 was ectopically expressed in HEK293T cells, immunoprecipitated, digested by proteases and subjected to LC-MS/MS analysis. Five peptides were identified harboring 3 unambiguously detected acetylation sites: K264, K274 and K275 (Fig. 1A, B). In addition, cells were supplemented with histone deacetylase (HDAC) inhibitors nicotinamide (NAM) and Trichostatin A (TSA), which inhibit endogenous deacetylase activity. Two additional unambiguously identified acetylation sites were detected: K6 and K259 (Fig. 1B). These observations indicate that FOXA2 can be acetylated on at least 5 residues.

The LC-MS/MS analyses identified FOXA2 peptides covering 81% of the whole FOXA2 sequence (data not shown). Although it cannot be excluded that acetylation sites were missed, the MS analyses identified 15 out of all 19 lysine residues, with each lysine covered several times. Next, the position of the acetylated sites was mapped on the FOXA2 structure which revealed that they were localized adjacent to the Forkhead domain (Fig. 1C).

### Deacetylation of FOXA2 is mediated by SIRT1

**Figure 2:**
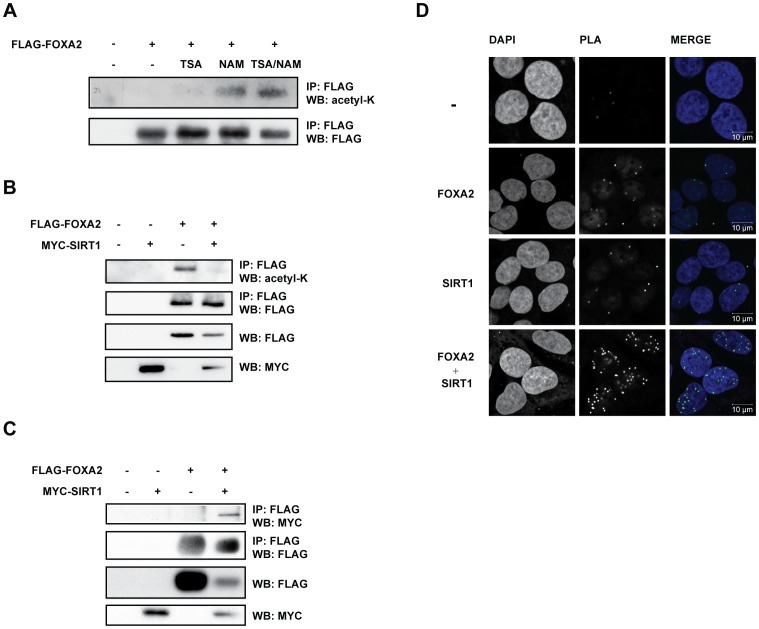
SIRT1 interacts with and deacetylates FOXA2. (A) Increase in FOXA2 acetylation by NAM. FLAG-FOXA2-transfected HEK293T cells were treated with class I/II HDAC inhibitor TSA (5 µM), class III HDAC inhibitor NAM (20 mM) or both for 16 hours. Acetylation level of immunoprecipitated FLAG-FOXA2 was assessed on Western Blot probed with an anti-acetylated-lysine specific antibody. (B) SIRT1 co-transfection affects FOXA2 acetylation. HEK293T cells were transfected with FLAG-FOXA2 alone or together with MYC-SIRT1 in the presence of 20 mM NAM. Acetylation was assessed as in (A). (C) SIRT1 interacts with FOXA2. HEK293T cells were co-transfected as in (B) followed by co-immunoprecipitation with anti-FLAG M2 beads. Interaction of FLAG-FOXA2 and MYC-SIRT1 was assessed by Western Blot analysis with indicated antibodies. (D) Endogenous FOXA2 and SIRT1 interact in the nucleus. HepG2 cells were subjected to immunofluorescence-based proximity ligation assay [28]. Nuclei were counterstained with DAPI.

The LC-MS/MS experiments revealed acetylated residues in FOXA2 upon treatment with HDAC inhibitors (Fig. 1B), suggesting that FOXA2 is a substrate for HDAC-mediated deacetylation. To determine which family of HDACs is responsible for deacetylation of FOXA2, FLAG-FOXA2 was ectopically expressed in HEK293T cells which were incubated with TSA (inhibitor of class I and II HDACs) and/or NAM (inhibitor of class III NAD^+^-dependent deacetylases, or sirtuins). Using a pan-acetylation-specific antibody (anti-acetyl-K) we observed that FOXA2 was robustly acetylated in cells treated with NAM, but not in cells with TSA alone (Fig. 2A). These data suggest that FOXA2 is a sirtuin-dependent deacetylation substrate.

To investigate whether SIRT1 is capable of deacetylating FOXA2, FLAG-FOXA2 and MYC-SIRT1 were co-expressed in NAM-treated HEK293T cells. Similar as in Fig. 2A, FOXA2 was robustly acetylated in the presence of NAM (Fig. 2B). Ectopic expression of SIRT1 overcame the NAM-mediated inhibition of endogenous sirtuin activity and completely blunted acetylation of FOXA2. These data therefore suggest that FOXA2 is a substrate for SIRT1-mediated deacetylation.

### SIRT1 interacts with and co-localizes with FOXA2 in the nucleus

Next, we assessed whether SIRT1-mediated deacetylation of FOXA2 occurs via a direct interaction of both proteins. Indeed, upon ectopic expression in HEK293T cells, MYC-SIRT1 co-immunoprecipitated with FLAG-FOXA2 suggesting that both proteins interact (Fig. 2C). To further examine the SIRT1-FOXA2 interaction, we performed a Proximity Ligation Assay [28], an immunofluorescence-based method that allows for intracellular detection of two proteins that are in close proximity of each other [29]. A PLA assay performed on the human hepatocellular carcinoma cell line HepG2, which endogenously expresses FOXA2 and SIRT1, showed interaction of these proteins (Fig. 2D). This interaction was strictly observed in the nucleus, which is in agreement with the reported nuclear activity of SIRT1 [30]. These findings were corroborated upon ectopic expression of MYC-SIRT1 and FLAG-FOXA2 in human osteosarcoma U2OS cells (which do not express FOXA2 endogenously), as we found a specific interaction of FLAG-FOXA2 and MYC-SIRT1 by using FLAG- and SIRT1-specific antibodies (data not shown). Collectively, these data show that SIRT1 and FOXA2 interact at an endogenous level exclusively in the nucleus.

### Interaction of FOXA2 and SIRT1 is reduced upon nutrient starvation

**Figure 3:**
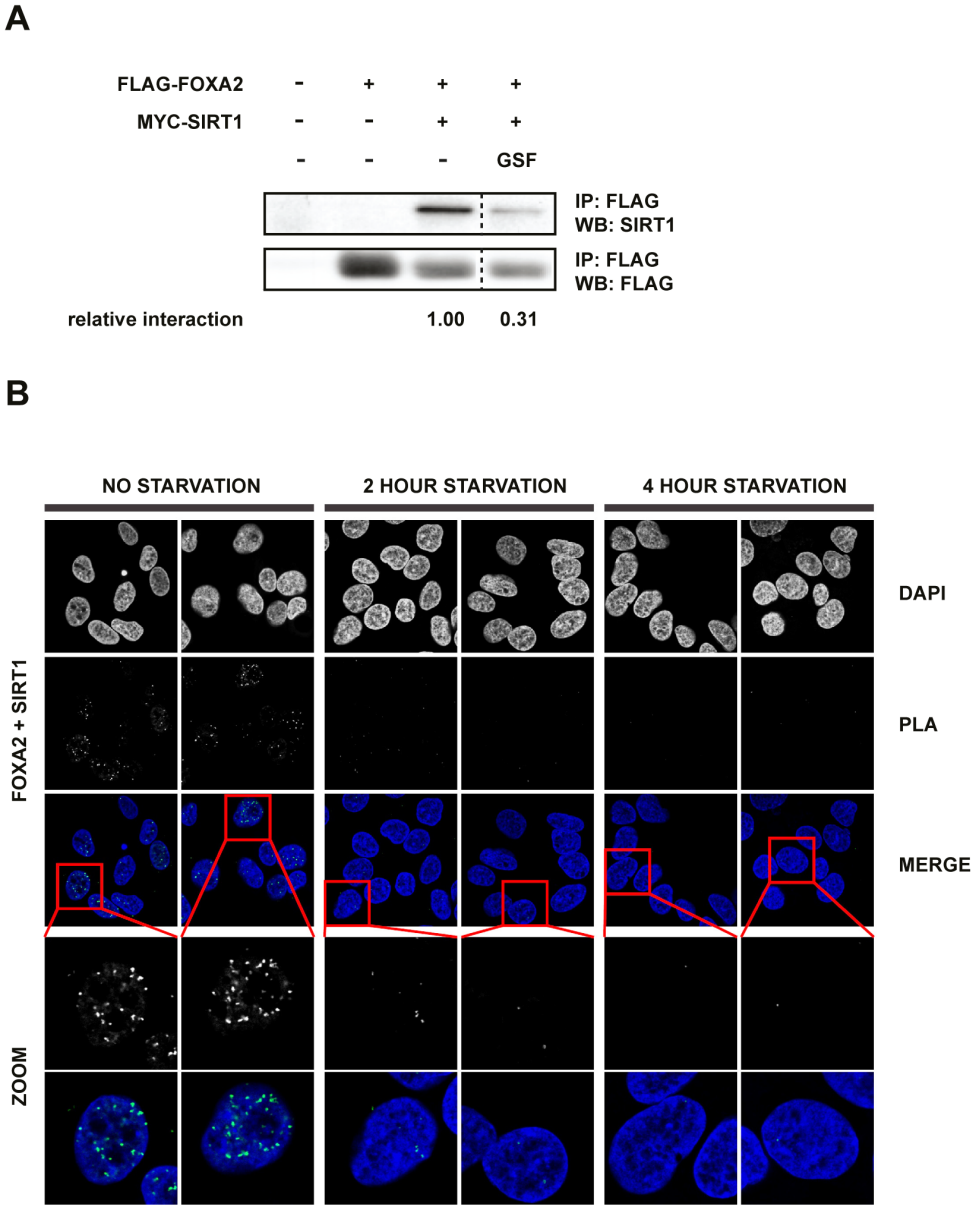
Nutrient withdrawal reduces SIRT1 and FOXA2 interaction. (A) Nutrient withdrawal reduced SIRT1-FOXA2 interaction. HEK293T cells were co-transfected with indicated constructs and starved from glucose and serum for 16 hours. FOXA2 and SIRT1 were co-immunoprecipitated with anti-FLAG-M2 beads and protein levels were assessed by Western Blot analysis probed with the indicated antibodies. Relative interaction is defined as the fraction of FOXA2 that binds to SIRT1. (B) Reduced endogenous interaction of FOXA2 and SIRT1 in the nucleus upon nutrient withdrawal as in A for 2 or 4 hours. Interaction between endogenously expressed SIRT1 and FOXA2 was assessed in HepG2 cells by PLA. Nuclei were counterstained with DAPI.

Because SIRT1 functions as a NAD^+^-dependent energy sensor we wondered whether the interaction of SIRT1 with FOXA2 is nutrient sensitive. To assess this, HEK293T cells, which ectopically expressed FLAG-FOXA2 and MYC-SIRT1, were subjected to nutrient stress for 16 hours. Strikingly, when compared to cells grown in standard medium, the interaction between FLAG-FOXA2 and MYC-SIRT1 was decreased upon starvation (deprivation of glucose and serum) (Fig. 3A). These findings were corroborated by observations from a PLA assay, which revealed that the interaction of endogenous FOXA2 and SIRT1 in HepG2 cells was reduced upon nutrient starvation (Fig. 3B). Collectively, these data indicate that the interaction of SIRT1 with FOXA2 is dependent on environmental nutrient levels.

### SIRT1 inhibits FOXA2-driven transcription

**Figure 4:**
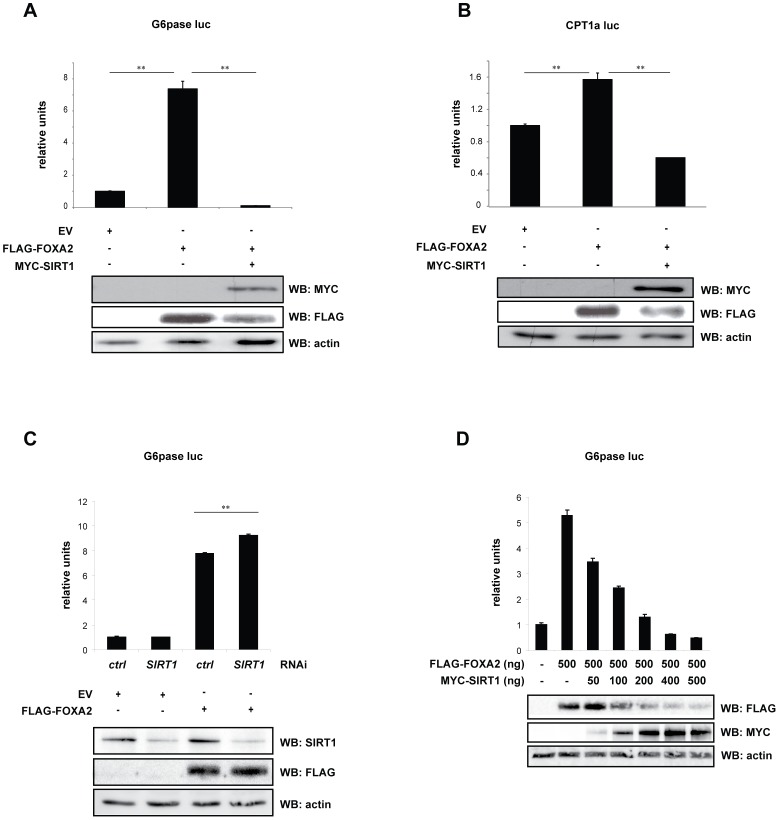
SIRT1 inhibits FOXA2-driven transcription. (A) and (B) SIRT1 co-transfection affects the FOXA2 transcriptional program. HEK293T cells were co-transfected with the indicated constructs and TK *Renilla* in combination with (A) *G6pase* and (B) *CPT1a* reporter constructs. After 48 hours, a luciferase-driven transcriptional assay was performed to assess FOXA2-mediated transcriptional activity on the promoter constructs. (C) SIRT1 knock-down increased FOXA2-driven transcription on the *G6pase* gene promoter. HEK293T cells were transfected with either control, or SIRT1 siRNA and subsequently co-transfected as in (A). Transcriptional activity was assessed via luciferase assays. (D) SIRT1 affects FOXA2-driven transcription by reducing FOXA2 protein levels. HEK293T cells were transfected as in (A) with FLAG-FOXA2 alone or in combination with increasing amounts of MYC-SIRT1. Empty pcDNA3 vector was co-transfected to balance between samples for the amount of DNA transfected. Transcriptional analysis was determined by luciferase assays. Values shown represent the mean of three independent experiments +SEM. Statistical analysis was performed by two-tailed Student's *t*-Test. **, *P*<0.01. Protein levels were assessed by Western Blotting using indicated antibodies. Actin was used as loading control.

FOXA2 is involved in regulating transcription of enzymes involved in glucose and lipid homeostasis, such as glucose-6-phosphatase (G6pase) and carnitine palmitoyltransferase 1a (CPT1a), respectively [1], [3]. To establish whether FOXA2-driven transcription is affected in a SIRT1-dependent manner, we performed a luciferase-driven transcription assay on *G6pase* and *CPT1a* promoter constructs. In the presence of SIRT1, FOXA2-driven transcription is blunted on both promoters (Fig. 4A, 4B). Conversely, knockdown of endogenous SIRT1 in HEK293T cells by siRNA revealed enhanced FOXA2-mediated transcription on the *G6pase* promoter (Fig. 4C). Moreover, a progressive decrease in FOXA2-driven transcription on the *G6pase* promoter was observed which was inversely proportional to the amount of co-transfected MYC-SIRT1 (Fig. 4D), indicating that SIRT1 affects the FOXA2 transcriptional program in a dose-dependent manner. Collectively, these data demonstrate that SIRT1 can inhibit FOXA2-driven transcription. Remarkably, expression analysis revealed that FLAG-FOXA2 protein levels decreased concomitantly with increasing amounts of co-transfected MYC-SIRT1 (Fig. 4D) suggesting SIRT1 negatively affects FOXA2-driven transcription by reducing FOXA2 protein levels, thereby modulating its transcriptional output.

### SIRT1-mediated deacetylation reduces FOXA2 protein stability by targeting it towards proteasomal degradation

**Figure 5:**
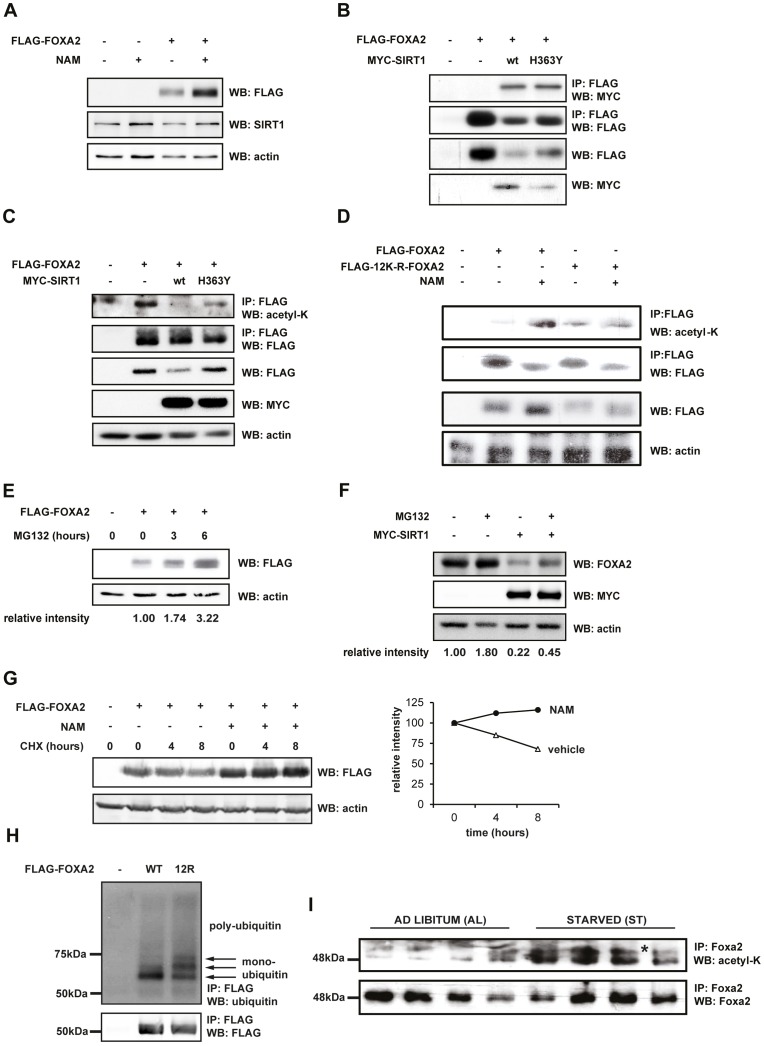
SIRT1-mediated deacetylation targets FOXA2 towards proteasomal degradation. (A) SIRT1 affects FOXA2 protein stability. HEK293T cells were transfected with FLAG-FOXA2 in the presence of 20 mM NAM or vehicle for 16 hours prior to cell lysis. Western Blots were probed for FLAG, and actin as a loading control. (B) Wild type SIRT1 and mutant SIRT1 H363Y similarly interact with FOXA2. HEK293T cells were transfected with FLAG-FOXA2 alone or in combination with either wild-type (wt) or the catalytically impaired MYC-SIRT1-H363Y mutant. FOXA2 and SIRT1 were co-immunoprecipitated with anti-FLAG-M2 beads and protein levels were assessed by Western Blot analysis probed with the indicated antibodies. (C) SIRT1 catalytic activity regulates FOXA2 stability. HEK293T cells were transfected and subjected to co-immunoprecipitation as in (B). Protein expression and FOXA2 acetylation level were assessed by Western Blot using the indicated antibodies. (D) Acetylation-impaired FLAG-12K-R-FOXA2 mutant showed reduced acetylation and protein stability. HEK293T cells were transfected with either FLAG-FOXA2 or FLAG-12K-R-FOXA2 and cultured as in (A). After FLAG-immunoprecipitation, protein levels were assessed by Western Blot probed with the indicated antibodies. (E) FOXA2 is subjected to proteasomal degradation in a time-dependent manner. HEK293T cells were transfected with FLAG-FOXA2 and supplemented with proteasome inhibitor MG132 (20 µM) or vehicle-containing medium for 3 or 6 hours. FOXA2 protein levels were assessed by Western Blot, and actin as a loading control. (F) SIRT1 mediates endogenous FOXA2 breakdown. HepG2 cells were transfected with MYC-SIRT1 and supplemented with 20 µM MG132- or vehicle-containing medium for 3 hours. (G) NAM preserves stability of FOXA2 upon abrogation of protein translation. HEK293T cells were transfected with FLAG-FOXA2 and supplemented 20 mM NAM or vehicle for 16 hours, followed by addition of cycloheximide (5 µg/ml) or vehicle-containing medium for 4 or 8 hours. (E–G) show a representative experiment from three independent experiments, and quantification of FOXA2 protein levels was performed by normalizing to actin protein levels. (H) 12K-R FOXA2 mutant has a higher level of poly-ubiquitylation. HEK293T cells were transfected with FLAG-FOXA2 or FLAG-12K-R-FOXA2. After culture of 20 µM MG132 for 3 hours, FOXA2 was immunoprecipitated with FLAG-M2 beads and immunoprecipitated proteins were assessed by Western Blot probed with the indicated antibodies. (I) Nutrient-dependent regulation of Foxa2 acetylation level in murine livers. Murine Foxa2 was immunoprecipitated from whole liver protein lysates from mice fed *ad libitum* (AL) or starved (ST) to 25% of *ad libitum* levels. Western Blots were probed with the indicated antibodies. The asterisk (*) marks a co-eluting acetylated protein of unknown identity.

To further investigate whether endogenous SIRT1 is able to affect FOXA2 protein levels, FLAG-FOXA2 was ectopically expressed in HEK293T cells. Inhibition of endogenous SIRT1 activity by NAM resulted in enhanced protein levels of FOXA2 whereas *FOXA2* mRNA levels remained unaffected (Fig. S1), suggesting that sirtuin-controlled FOXA2 acetylation may be important for FOXA2 protein stability (Fig. 5A).

The deacetylation activity of SIRT1 is mediated by a critical histidine residue (H363) located in its NAD^+^-binding catalytic site [31]. To investigate whether the acetylation state and concomitant decrease in FOXA2 protein levels is dependent on the catalytic activity of SIRT1, either wild type SIRT1 or the SIRT1-H363Y catalytically-impaired mutant were co-expressed with FLAG-FOXA2 in HEK293T cells. FOXA2 was found to interact with both SIRT1 and SIRT1-H363Y (Fig. 5B), indicating that the interaction is independent of SIRT1 activity. However, in contrast to wild type SIRT1, expression of SIRT1-H363Y reduced FOXA2 protein levels less severely, as FOXA2 expression was 67% (Fig. 5B) and 142% (Fig. 5C) higher upon co-transfection with SIRT1-H363Y than with wild type SIRT1). In addition, acetylation of FOXA2 was predominantly retained upon SIRT1-H363Y co-expression (Fig. 5C). Hence, the NAD^+^-dependent catalytic activity of SIRT1 appears to be required for the stability of the FOXA2 protein.

To identify which acetylated residue(s) are critical for stabilization of FOXA2, we iteratively mutated all the five identified lysines (K) to arginine (R) residues. These mutant FOXA2 proteins were ectopically expressed in HEK293T cells and endogenous SIRT1 activity was inhibited with NAM. When the 5 acetylation sites identified by our LC-MS/MS analysis (Fig. 1B) were mutated it was noted that protein expression levels were slightly reduced as compared to wild type FOXA2, however a significant level of acetylation was still observed (data not shown). This suggests that other acetylated lysines must be present in FOXA2 that are controlled in a sirtuin-dependent manner, and which were not uncovered by our LC-MS/MS analysis. To investigate this hypothesis, a FLAG-12K-R-FOXA2 mutant was generated, in which 12 out of 19 FOXA2 lysine residues were changed to arginine. K365 and K399 were additionally mutated since these residues were not covered in our LC-MS/MS analyses, K149 was found to be acetylated but with a non-significant peptide score (data not shown), while K226, K229, K253 and K256, although not identified as acetylated by the LC-MS/MS approach, could be potential targets of histone acetyltransferase p300 since they make up the -K-X-X-K- consensus motif of p300 [32]. Ectopic expression in HEK293T cells showed a marked reduction of FLAG-12K-R-FOXA2 protein levels compared to wild type FOXA2 (Fig. 5D). In contrast to 12K-R-FOXA2 protein levels, wild type FOXA2 expression was stabilized by NAM and the acetylation level of FOXA2 was enhanced. Although 12K-R-FOXA2 showed a low basal level of acetylation, little increase was observed when cells were treated with NAM (Fig. 5D). Collectively, these data indicate that these 12 lysines are critical for the SIRT1-mediated regulation of FOXA2 acetylation levels.

Next, we investigated whether the SIRT1-mediated decrease in FOXA2 protein levels is the result of proteasomal degradation of FOXA2. Therefore, FLAG-FOXA2 was transfected in HEK293T cells and treated with the proteasome inhibitor MG132 for 3 and 6 hours. Similar to treatment with NAM, FOXA2 protein levels were increased upon MG132 treatment, suggesting that FOXA2 is subject to degradation in the proteasome (Fig. 5E). Moreover, in HepG2 cells, the observed reduction in endogenous FOXA2 protein levels resulting from MYC-SIRT1 expression was partly abolished upon MG132 treatment (Fig. 5F), suggesting that SIRT1-mediated deacetylation of FOXA2 leads, at least in part, to FOXA2 degradation in the proteasome. Subsequently, we blocked protein translation in HEK293T cells with cycloheximide and found that protein levels of ectopically-expressed wild type FOXA2 decreased substantially within 8 hours after cycloheximide addition. In contrast, prior treatment with NAM abrogated the decrease in FOXA2 protein levels induced by cycloheximide (Fig. 5G). Collectively, these data show that acetylation of FOXA2 protects the protein from degradation.

As FOXA2 is degraded at least in part by the proteasome, we wondered whether the protein is prone to ubiquitination. Therefore, we immunoprecipitated ectopically expressed wild-type or 12K-R FLAG-FOXA2 from HEK293T cells, blocked proteasomal degradation, and assessed the level of FOXA2 ubiquitylation. We found that the 12K-R mutant showed an increased level of poly-ubiquitination than wild-type FOXA2 (Fig. 5H). Of note, we observed that FOXA2 was also mono-ubiquitinated at three sites (Fig. 5H; arrows), and that the level of mono-ubiquitination was increased in the 12K-R mutant. These data at least suggest that the higher level of ubiquitination of the 12K-R deacetylation-mimicking mutant results in reduced protein stability by targeting the protein towards degradation in the proteasome.

### Nutrient deprivation enhances acetylation of Foxa2 in murine livers

FOXA2 is predominantly expressed in metabolically active tissues such as liver and pancreas. To test if the nutrient-dependent acetylation of FOXA2 also occurs *in vivo*, we assessed Foxa2 acetylation levels in livers from mice fed with either a isocaloric diet (*ad libitum* fed, AL) or which had a diet consisting of 25% of *ad libitum* levels (starved, ST) for 36 hours. Endogenously expressed Foxa2 was immunoprecipitated from liver homogenates and acetylation levels were assessed on Western Blot with a pan-acetylation-specific antibody. Acetylation levels of Foxa2 in livers from ST mice were increased as compared to those in livers of AL fed mice (Fig. 5I), revealing that Foxa2 is regulated by acetylation in a nutrient-dependent manner *in vivo*.

## Discussion

Mammalian Foxa2 regulates glucose and lipid homeostasis in the liver [3]–[7] in response to fasting, but how this transcription factor is controlled and how it relays signals in response to extracellular cues, is not completely understood. The present study provides evidence for nutrient-dependent post-translational regulation of FOXA2 via acetylation which is negatively regulated by the sirtuin deacetylase family member SIRT1. By means of LC-MS/MS analyses, we identified 5 acetylated lysine residues on FOXA2 and found that SIRT1 interacts with and deacetylates FOXA2 in a nutrient-dependent manner. Importantly, upon deacetylation by SIRT1, FOXA2 becomes susceptible to proteasomal degradation by enhanced mono- and poly-ubiquitination, hence affecting the FOXA2 transcriptional program due to reduced FOXA2 protein levels. We confirmed these observations *in vivo* by showing that acetylation levels of Foxa2 in mouse liver were increased upon nutrient starvation, likely resulting from reduced interaction between Foxa2 and Sirt1. Therefore, we propose that acetylation of FOXA2 serves as a rapid mechanism to prevent degradation of FOXA2, when it is needed to regulate metabolic homeostasis in times of nutrient shortage. In addition, these data suggest that FOXA2-mediated transcriptional regulation of metabolism is directly controlled by SIRT1 upon changes in intracellular energy levels.

In our LC-MS/MS analyses, 3 of the 5 identified acetylated lysine residues were found to be specifically present in FOXA2, whereas the conserved K6 and K259 were also found acetylated in FOXA1. For example, lysine K6 is conserved among all FOXA members and indeed we identified this residue acetylated on FOXA1 as well (data not shown). Interestingly, Belagouli et al. [33] reported that stability of FOXA2 is regulated by sumoylation on residue K6. The metabolic cofactor CRTC2 has recently been shown to be deacetylated by SIRT1 and then subjected to sumoylation-driven breakdown [34]. It is therefore attractive to speculate that competition between these modifications on K6 may regulate protein levels of FOXA2 in response to SIRT1. However, K6 itself seemed not essential to regulate SIRT1-mediated FOXA2 stability at least in kidney cells, as mutation of this residue to arginine (K to R) showed relatively similar FOXA2 protein and acetylation levels as the wild type protein (data not shown). It is important to note that mutation of K6 also renders the protein unsusceptible to sumoylation and therefore further research is needed to determine whether any interplay between sumoylation and acetylation on FOXA2 K6 occurs in other cell types.

We found that mutation of at least twelve lysines was required to reduce the overall acetylation signal on FOXA2. Of note, some of the mutated lysines may only represent putative acetylation sites for example due to low stoichiometry and as a result these may not be identified by LC-MS/MS. Some lysines in FOXA2 were not covered by LC-MS/MS despite various digestion strategies, and therefore further confirmation is needed to dissect whether other than the identified residues in FOXA2 are acetylated. Nevertheless, the acetylation sites that were uncovered by our LC-MS/MS analyses play an important role in regulating FOXA2 protein stability.

In our study, FOXA2 was found to be both mono- and poly-ubiquitinated. Poly-ubiquitination is primarily regarded as a signal that enables transport of the protein to the proteasome for degradation. This notion is in line with our observation that breakdown of FOXA2, mediated by SIRT1 is MG132 sensitive and suggests that at least part of the FOXA2 degradation is mediated by the proteasome. Mono-ubiquitination, however, can impact proteins in various ways and has been described to affect cellular events including DNA repair, gene expression, endocytosis and nuclear export [35]. However, it has also been demonstrated that mono-ubiquitination can also provide a signal for lysosomal degradation of proteins [36]. Although further study is required to determine the exact role for the three mono-ubiquitinated sites in FOXA2, the increase in the level of mono-ubiquitination in the 12K-R mutant may imply their involvement in breakdown of FOXA2.

In agreement with our data, a recently published study by Von Meyenn *et al*. [37] elegantly showed K259 and K275 as acetylated residues in Foxa2. Acetylation of K259 was induced by p300 by glucagon-mediated signaling during fasting, resulting in expression of genes involved in β-oxidation and ketone body formation. Von Meyenn *et al*. also showed that SIRT1 is able to deacetylate Foxa2. The work reported here corroborates these findings and goes on to add a novel dimension to these observations. Rather than observing effects of acetylation on Foxa2 shuttling, we observe that SIRT1 affects FOXA2 transcriptional output in a nutrient-dependent manner by means of regulating FOXA2 protein stability. The nuclear shuttling of Foxa2 in an insulin-dependent manner reported by the Stoffel group [3], [15] remains controversial as we (data not shown) and others have not found any evidence for this, which has been discussed elsewhere in detail [6], [13]. However, our observation that SIRT1 mediates stability of FOXA2 protein levels does not exclude the nuclear shuttling model, but in contrast represents another layer of regulation of FOXA2 by means of acetylation to execute the appropriate metabolic program during nutrient limitation. Taken together, the maintenance of FOXA2 expression and function by acetylation seems dually regulated during nutrient deprivation. On the one hand, glucagon signaling enhances p300 acetyltransferase activity to induce acetylation of FOXA2. On the other hand, the reduced interaction of SIRT1 and FOXA2 enhances acetylation of FOXA2 by the loss of SIRT1-mediate deacetylation of the protein. It remains unclear how the FOXA2-SIRT1 interaction is reduced upon nutrient deprivation. Possibly alterations in other post-translational modifications, e.g. phosphorylation of either FOXA2 or SIRT1, may reduce the interaction between both proteins. Alternatively, enhanced activation of p300 (increased phosphorylation of Ser89) upon glucagon signaling may compete with SIRT1 for access to the acetylation sites on FOXA2 [34]. Further research on the FOXA2-SIRT1 interaction is required to fully understand this nutrient dependent regulation of FOXA2.

The work reported here also shows additional differences compared to the work presented by Von Meyenn et al. Firstly, Von Meyenn et al. studied murine Foxa2 and showed that mutation of K259 and K275 residues to arginine completely blunted overall Foxa2 acetylation in Hepa1-6 cells. Secondly, pharmacological inhibition by NAM alone was not sufficient to increase Foxa2 K259 acetylation levels, and inhibition of HDACs was additionally required. In contrast, we report here the requirement of at least 12 mutated lysines for reduction of the acetylation signal of FOXA2, and a robust induction of acetylation by NAM treatment alone. Moreover, we identified three additional acetylation sites in FOXA2 when ectopically expressed in HEK293T cells. These differences could be related to species differences of the cell types and constructs that were used. This notion is supported by von Meyenn *et al*. as they show that only acetylation of one of the two identified acetylation sites (K259) in murine Foxa2 was found in human HepG2 cells whereas both K259 and K275 were found in mouse Hepa1-6 cells [37]. Moreover, the level of coverage likely contributes as 81% of FOXA2 was covered by LC-MS/MS in our study as compared to 37% and 57% of Foxa2 in the study of Von Meyenn et al. Indeed, we identified acetylation on K6, a residue that was not covered by Von Meyenn et al.

**Figure 6:**
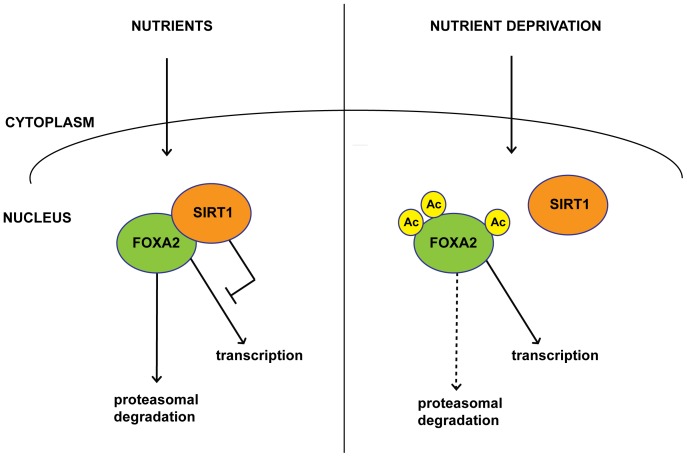
Model for nutrient-dependent regulation of FOXA2 stability via SIRT1-mediated deacetylation. Graphical representation of the interaction and deacetylation of FOXA2 by SIRT1 depending on available nutrient levels. In times of nutrient excess (left panel) SIRT1 interacts with FOXA2 in the nucleus, resulting in FOXA2 deacetylation and subsequent proteasomal degradation. When nutrients are limited (right panel), FOXA2 is required to exert its transcriptional program in the nucleus. The interaction of FOXA2 with SIRT1 is diminished, resulting in enhanced acetylation and stability of FOXA2.

In conclusion, we propose a model in which FOXA2 protein levels are regulated by SIRT1 via acetylation-mediated proteasomal degradation (Fig. 6). In the presence of nutrients, FOXA2 is dispensable for metabolic homeostasis and therefore the balance is towards a deacetylated state, mediated by SIRT1, resulting in enhanced degradation of FOXA2 in the proteasome. Conversely, in times of nutrient stress, FOXA2 needs to be switched on to drive gluconeogenesis, β-oxidation and ketone body formation. FOXA2 is then released from the inhibitory interaction with SIRT1 by an, as of yet, unknown mechanism, thereby reducing FOXA2 deacetylation. In addition, glucagon signaling enhances p300 activity in a cAMP-PKA dependent manner to drive Foxa2 acetylation [34], [37]. Together with previous published work, our data provides better understanding of the molecular control of metabolism by SIRT1 and FOXA2, and therefore may contribute to the development of intervention strategies for metabolic derangements like diabetes or obesity.

## Supporting Information

Figure S1 NAM does not affect mRNA levels of FOXA2. HEK293T cells were transfected with FLAG-FOXA2 or empty vector. Cells were cultured with or without 20 mM NAM for 16 hours, after which cells were lysed, RNA was isolated and mRNA levels of *FOXA2* were determined by quantitative RT-PCR. *18 S* mRNA levels were used as a reference. Results depict mean+SEM from three independent experiments.
(TIF)
