# Supplementary figures and images for: SIRT1 Mediates FOXA2 Breakdown by Deacetylation in a Nutrient-Dependent Manner
Source: PLoS One. 2014 May 29;9(5):e98438. doi: 10.1371/journal.pone.0098438 (PMC4038515; doi:10.1371/journal.pone.0098438)

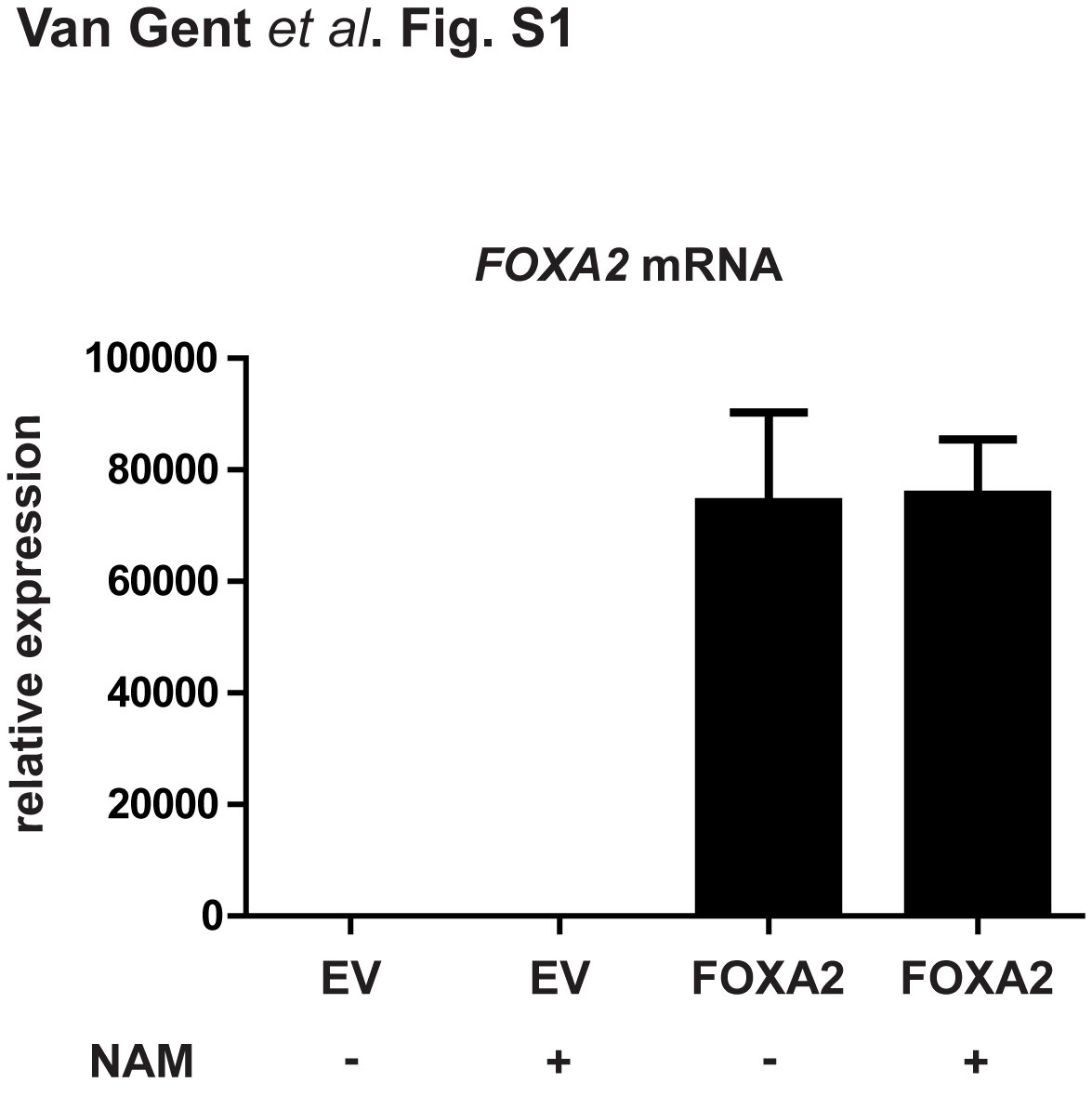

Supplement: Figure S1 — NAM does not affect mRNA levels of FOXA2. HEK293T cells were transfected with FLAG-FOXA2 or empty vector. Cells were cultured with or without 20 mM NAM for 16 hours, after which cells were lysed, RNA was isolated and mRNA levels of FOXA2 were determined by quantitative RT-PCR. 18 S mRNA levels were used as a reference. Results depict mean+SEM from three independent experiments. (TIF) [file pone.0098438.s001.tif]
